# Predictive factors for exacerbation and re-exacerbation in chronic obstructive pulmonary disease: an extension of the Cox model to analyze data from the Swiss COPD cohort

**DOI:** 10.1186/s40248-019-0168-5

**Published:** 2019-02-05

**Authors:** Pascal Urwyler, Nebal Abu Hussein, Pierre O. Bridevaux, Prashant N. Chhajed, Thomas Geiser, Peter Grendelmeier, Ladina Joos Zellweger, Malcolm Kohler, Sabrina Maier, David Miedinger, Michael Tamm, Robert Thurnheer, Thomas Dieterle, Joerg D. Leuppi

**Affiliations:** 1 University Clinic of Medicine, Cantonal Hospital Baselland, University of Basel, Rheinstrasse 26, 4410 Liestal, Switzerland; 2 University Hospital Basel, University of Basel, Spitalstrasse 21, 4031 Basel, Switzerland; 3 Hospital of Valais, University of Geneva, Avenue du Grand-Champsec 80, 1950 Sion, Switzerland; 4 University Hospital Bern (Inselspital), University of Bern, Freiburgstrasse 18, 3010 Bern, Switzerland; 5 St. Clara Hospital, University of Basel, Kleinriehenstrasse 30, 4002 Basel, Switzerland; 6 University Hospital Zurich, University of Zurich, Rämistrasse 100, 8091 Zürich, Switzerland; 7 Cantonal Hospital of Muensterlingen, Spitalcampus 1, 8596 Münsterlingen, Switzerland

**Keywords:** COPD, Exacerbation, Re-exacerbation, Primary health care, Risk factors

## Abstract

**Background:**

The Swiss COPD cohort was established in 2006 to collect data in a primary care setting. The objective of this study was to evaluate possible predictive factors for exacerbation and re-exacerbation.

**Methods:**

In order to predict exacerbation until the next visit based on the knowledge of exacerbation since the last visit, a multistate model described by Therneau and Grambsch was performed.

**Results:**

Data of 1,247 patients (60.4% males, 46.6% current smokers) were analyzed, 268 (21.5%) did not fulfill spirometric diagnostic criteria for COPD. Data of 748 patients (63% males, 44.1% current smokers) were available for model analysis. In order to predict exacerbation an extended Cox Model was performed. Mean FEV_1_/FVC-ratio was 53.1% (±11.5), with a majority of patients in COPD GOLD classes 2 or 3. Hospitalization for any reason (HR1.7; *P* = 0.04) and pronounced dyspnea (HR for mMRC grade four 3.0; *P* < 0.001) at most recent visit as well as prescription of short-acting bronchodilators (HR1.7; *P* < 0.001), inhaled (HR1.2; *P* = 0.005) or systemic corticosteroids (HR1.8; *P* = 0.015) were significantly associated with exacerbation when having had no exacerbation at most recent visit. Higher FEV_1_/FVC (HR0.9; *P* = 0.008) and higher FEV_1_ values (HR0.9; *P* = 0.001) were protective. When already having had an exacerbation at the most recent visit, pronounced dyspnea (HR for mMRC grade 4 1.9; *P* = 0.026) and cerebrovascular insult (HR2.1; *P* = 0.003) were significantly associated with re-exacerbation. Physical activity (HR0.6; *P* = 0.031) and treatment with long-acting anticholinergics (HR0.7; *P* = 0.044) seemed to play a significant protective role. In a best subset model for exacerbation, higher FEV_1_ significantly reduced and occurrence of sputum increased the probability of exacerbation. In the same model for re-exacerbation, coronary heart disease increased and hospitalization at most recent visit seemed to reduce the risk for re-exacerbation.

**Conclusion:**

Our data confirmed well-established risk factors for exacerbations whilst analyzing their predictive association with exacerbation and re-exacerbation. This study confirmed the importance of spirometry in primary care, not only for diagnosis but also as a risk evaluation for possible future exacerbations.

**Trial registration:**

Our study got approval by local ethical committee in 2006 (EK Nr. 170/06) and was registered retrospectively on ClinicalTrials.gov (NCT02065921, 19^th^ of February 2014).

## Background

Chronic obstructive pulmonary disease (COPD) is a respiratory disease, characterized by irreversible airflow limitation and one of the most deadly, prevalent, and costly chronic diseases [1]. COPD is known to be a progressive disease affecting more than 5% of the entire population [2, 3]. COPD is the underlying cause for significant morbidity, it ranks 10^th^ on a worldwide ranking assessing disease burden by disability-adjusted life years (DALYs) with 27.7 DALYs [4]. According to the WHO health statistics of 2008, COPD was the fourth leading cause of mortality in 2004 and is projected to be the third leading cause of death in 2030 [5]. Like other chronic diseases, COPD causes a substantial human and economic burden to society [6]. The growing worldwide burden is considered to be mainly due to cigarette smoking, environmental and occupational factors and the aging population [7, 8].

In this context, the Swiss COPD cohort was established in 2006 to collect spirometric and other relevant data of COPD patients treated in a primary care setting and to facilitate research on the diagnosis, treatment and clinical course of COPD [9–11].

Acute exacerbations of COPD (AECOPD) are key events in COPD. Not only do exacerbations increase the economic burden for society [12], but they are also associated with a faster decline in lung function [13, 14], lower quality of life [15] and increased morbidity [16] and mortality [17]. Although AECOPD are an emerging field of research and several possible associated risk factors have been reported, they are still not clearly defined. So far, the greatest risk factor seems to be a previous history of exacerbations [18]. More recently, Make et al. introduced an easily applicable score to predict short-term risk of COPD exacerbations (SCOPEX) which includes the following factors: sex, number of COPD maintenance medications, number of exacerbations in the previous year, FEV_1_/FVC ratio and reliever use [19]. Almagro et al. described the CODEX index consisting of comorbidity assessment with Charlson index, airflow obstruction, dyspnea and previous severe exacerbations to predict survival and readmission after hospitalization for AECOPD [20]. With regards to re-exacerbation in patients with AECOPD, a recent study found age, lung function impairment, frequency of AECOPD during the previous year and some parameters of current AECOPD (such as pleural effusion, use of accessory respiratory muscles, medication and hospitalization length) to have a strong predictive capacity [21]. Another study showed an association between prolonged symptomatic duration of AECOPD and poorer health status with the risk of developing a new event [22]. In the primary care setting, however, only sparse data about potential risk factors for exacerbations and re-exacerbations are currently available [23].

The aims of this study were to analyze data of the entire patient population of the Swiss COPD cohort in a descriptive way and secondly, to evaluate known risk factors and their predictive association with exacerbation and re-exacerbation within a multi-state model framework previously reported by Therneau and Grambsch [24, 25].

## Methods

### Study design and patient population

We received ethical approval for this questionnaire-based observational COPD cohort study by the local ethical committee in 2006 (EK Nr. 170/06) and subsequently by ethical committees of all other participating Swiss cantons. A generic letter was sent to 225 GPs in 23 Swiss cantons and an initial total of 139 GPs agreed to participate. The study was also registered on ClinicalTrials.gov (NCT02065921).

All participating patients provided written informed consent. COPD patients treated in primary care were clinically evaluated at least every six months by their GPs. Spirometry was performed using an EasyOne™ spirometer (n.d.d. Medizintechnik AG, Zurich, Switzerland). Initially, all participating GPs were instructed how to perform spirometry in accordance with the guidelines of the American Thoracic Society and the European Respiratory Society [26, 27]. The Swiss reference values by Brändli et al. were used to calculate predicted values [28, 29]. In accordance with GOLD-guidelines, airway obstruction was defined as presence of a FEV_1_/FVC < 70% after bronchodilation. A standardized questionnaire included spirometric parameters, as well as clinical and demographic data. All participating patients provided written informed consent.

**Fig. 1:**
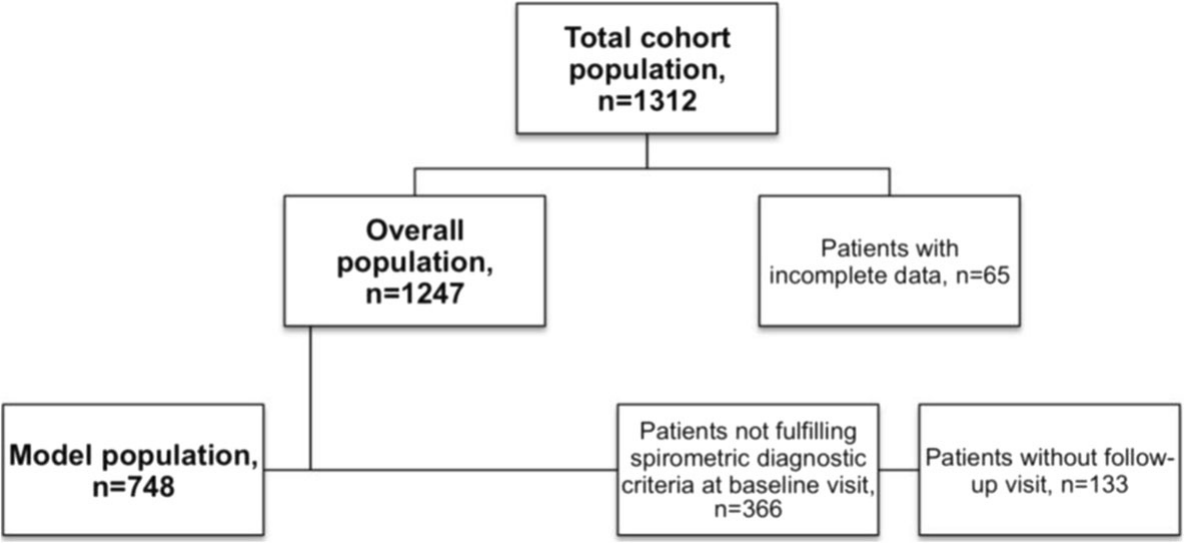
Study population

Details of patients excluded from the analysis are shown in Fig. 1. Until the end of 2014, 1,312 patients have been included in the study. After exclusion of 65 patients due to incomplete data, we analyzed data of a total of 1,247 patients (=overall population). A substantial amount of cohort patients with a clinical diagnosis of COPD did either not fulfill spirometric diagnostic criteria for COPD or had missing spirometric data (*n* = 366). After exclusion of patients who did not have at least one follow up visit, 748 patients were available for the model analysis (=model population).

### Collected data

Patients were seen at least every six months by their GPs. At baseline, demographic information such as age, sex, height, weight, year of diagnosis of COPD and risk factors (e.g. smoking status) was collected. Patients performed post-bronchodilatator lung function testing every six months and spirometric data (FEV_1_, FVC and FEV_1_/FVC ratio) was collected. At every visit, patients were asked about symptoms such as dyspnea (assessed for all patients with mMRC dyspnea scale), cough and sputum production. Information about COPD medication and non-pharmacological treatments was registered, as well as information about hospitalizations and exacerbations since the most recent visit. Exacerbation was defined as worsening of clinical symptoms leading to a change in treatment. Anonymized data of all patients was captured in a central electronic database.

### Statistical analysis

Descriptive statistics were performed presenting mean and standard deviation (SD) or count and percentage as appropriate. In order to compare categorical data between study groups, chi-square tests were used. In the case of continuous data T-tests were used.

In order to predict exacerbation until the next visit based on the knowledge of exacerbation since the most recent visit, a multistate model on the basis of a multi-state Cox model approach reported by Therneau and Grambsch [25] was performed. This model was used to describe a cohort of patients with Crohn’s disease in Olmsted County Minnesota [24]. The argumentation for using this model was based on some similar characteristics shared by Crohn’s disease and COPD: both are (i) chronic diseases with (ii) waxing and waning symptoms, which (iii) make it difficult to describe or predict long-term outcomes. Multistate models are appropriate in case of events (or states) that occur from time point to time point. In our case, the event is defined as “having an exacerbation since the most recent visit”. The following four state changes from visit to visit are possible: exacerbation to no exacerbation, exacerbation to exacerbation, no exacerbation to exacerbation, no exacerbation to no exacerbation.

Further details and examples are described in a book by Therneau and Grambsch [25].

For each state change potential predictors can be identified. Results are presented as hazard ratios (HR) with 95% confidence intervals for each predictor separately. Additionally, a multivariable best subset of predictors was selected using Akaike’s information criterion (AIC) [30]. No multivariable model including all available predictors was done, because the estimated HRs and *p* are often misleading and could be misinterpreted.

Results are expressed as HRs with corresponding 95% confidence intervals and *p*. For ordinal and continuous variables HRs were generally expressed increasing the predictor one unit. For age, FEV_1_, FVC, FEV_1_/FVC ratio and BMI, HRs were expressed increasing the predictor ten units.

A *p* < 0.05 was considered to indicate statistical significance. This study was exploratory, therefore *p* were not adjusted for multiple comparisons.

All evaluations were done using the statistical software R (R Core Team, 2015, R: a language and environment for statistical computing, R Foundation for Statistical Computing, Vienna, Austria).

## Results

### Baseline characteristics

Baseline characteristics of the overall and model population are summarized in Table 1. The majority in both populations were males. Fifty-three percent were ex-smokers and 47% current smokers. Mean follow up time of our overall population was 1.2 years (±0.8 years) and mean follow up time of the model population was 1.39 years (±0.68 years).

**Table 1:** Baseline characteristics of the overall and model population

	Overall population	Known	Model population	Known
General characteristics	*N (%)/Mean (±SD)*	*N (%)*	*N (%)/Mean (±SD)*	*N (%)*
N total	1247		748	
Age (years)	66.4 (±11.8)	1243 (99.7%)	67.3 (±11.6),	748 (100%)
Sex, males	752 (60.4%)	1245 (99.8%)	471 (63.0%)	748 (100%)
BMI (kg/m2)	26.5 (±5.5)	1129 (90.5%)	26.2 (±5.29)	696 (93.0%)
Current smokers	577 (46.6%)	1239 (99.4%)	328 (44.1%)	744 (99.5%)
Ex-smokers	662 (53.4%)	1239 (99.4%)	416 (55.9%)	744 (99.5%)
Lung function				
FEV_1_/FVC	59.0% (±15.6)	1149 (92.1%)	53.1% (±11.5)	748 (100%)
FEV_1_ %Ref	54.9% (±20.8)	1088 (87.2%)	49.5% (±17.4)	718 (96.0%)
FVC %Ref	69.6% (±19.3)	1099 (88.1%)	69.5% (±18.9)	718 (96.0%)
No COPD	268 (24.3%)	1101 (88.3%)	0 (0%)	718 (96.0%)
GOLD 1	40 (3.63%)	1101 (88.3%)	32 (4.46%)	718 (96.0%)
GOLD 2	351 (31.9%)	1101 (88.3%)	303 (42.2%)	718 (96.0%)
GOLD 3	323 (29.3%)	1101 (88.3%)	287 (40.0%)	718 (96.0%)
GOLD 4	119 (10.8%)	1101 (88.3%)	96 (13.4%)	718 (96.0%)
Symptoms				
mMRC dyspnea scale 0–1	610 (50.8%)	1201 (96.3%)	355 (49.0%)	725 (96.9%)
mMRC dyspnea scale 2	350 (29.1%)	1201 (96.3%)	219 (30.2%)	725 (96.9%)
mMRC dyspnea scale 3	199 (16.6%)	1201 (96.3%)	129 (17.8%)	725 (96.9%)
mMRC dyspnea scale 4	42 (3.50%)	1201 (96.3%)	22 (3.03%)	725 (96.9%)
Cough	864 (69.4%)	1245 (99.8%)	515 (68.9%)	747 (99.9%)
Sputum	731 (58.8%)	1243 (99.7%)	446 (59.7%)	747 (99.9%)
Pharmacological COPD treatment				
Short-acting bronchodilators	466 (37.5%)	1242 (99.6%)	294 (39.4%)	747 (99.9%)
Long-acting ß_2_-agonists (LABA)	355 (28.7%)	1237 (99.2%)	235 (31.5%)	745 (99.6%)
Long-acting muscarinic antagonists (LAMA)	509 (41.1%)	1238 (99.3%)	345 (46.2%)	746 (99.7%)
Inhaled corticosteroids (ICS)	270 (21.8%)	1237 (99.2%)	178 (23.8%)	747 (99.9%)
Inhaled combination therapy (LABA+ICS)	632 (50.9%)	1242 (99.6%)	390 (52.1%)	748 (100%)
Systemic corticosteroids	73 (5.89%)	1240 (99.4%)	42 (5.61%)	748 (100%)
Comorbidities				
Asthma	221 (20.6%)	1073 (86.0%)	130 (19.9%)	654 (87.4%)
Diabetes mellitus	141 (13.1%)	1074 (86.1%)	86 (13.1%)	655 (87.6%)
Hypertension	601 (55.8%)	1078 (86.4%)	366 (55.9%)	655 (87.6%)
Coronary heart disease	228 (21.2%)	1075 (86.2%)	148 (22.6%)	655 (87.6%)
Heart failure	159 (14.8%)	1073 (86.0%)	96 (14.7%)	654 (87.4%)
Cancer (other than lung cancer)	52 (4.85%)	1072 (86.0%)	36 (5.51%)	653 (87.3%)
Lung cancer	29 (2.70%)	1073 (86.0%)	16 (2.45%)	654 (87.4%)
Cerebrovascular Insult (CVI)	36 (3.36%)	1073 (86.0%)	24 (3.67%)	654 (87.4%)
Physical activity				
Sports (at least twice a week)	334 (28.2%)	1183 (94.9%)	200 (27.6%)	724 (96.8%)
Pulmonary rehabilitation	86 (7.01%)	1226 (98.3%)	55 (7.40%)	743 (99.3%)

With regard to lung function, mean FEV_1_/FVC-ratio was 59% for the overall and 53.1% for the model population. In both the overall and the model population, patients with GOLD 1 severity of airflow limitation were rather scarcely represented with 3.63 and 4.46% respectively. Most patients had GOLD 2 or 3 severity of airflow limitation.

Regarding dyspnea, 50.8% of the overall and 49.0% of the model population had few symptoms with an mMRC grade 0–1. Five hundred and ninety-one patients (49.2%) of the overall population and 370 patients (51.0%) of the model population had more symptoms with an mMRC grade 2 or higher. The majority of all patients complained of cough and sputum.

Short acting bronchodilators were prescribed in less than half of our cohort’s patients. Concerning long-acting inhaled medication (ß_2_-agonists or anticholinergics), inhaled combination therapy (long-acting ß_2_-agonists (LABA) and inhaled corticosteroids) and long-acting muscarinic antagonists (LAMA) were prescribed most commonly. Systemic steroids were rarely used (see Table 1). Inhaled corticosteroids only (without LABA or LAMA) were used in a very small minority (26 out of 1,072 patients (2.1%)).

Frequent comorbidities were high blood pressure in more than half, as well as coronary heart disease and asthma in a fifth of all patients. Other frequently detected comorbidities were diabetes mellitus and heart failure.

### Factors associated with exacerbation and re-exacerbation

Results of the univariate analysis to assess factors associated with the risk “having an exacerbation until next visit” and “having a re-exacerbation until next visit” are shown in Table 2 and are based on a multistate model described above.

**Table 2:** Factors associated with exacerbation and re-exacerbation, univariate analysis

	Factors associated with exacerbation			Factors associated with re-exacerbation		
General characteristics	HR	Lower/upper 95% confidence interval	*p*	HR	Lower/upper 95% confidence interval	*p*
Age (years)	1.084	0.977/1.203	0.127	1.168	0.977/1.396	0.088
Male sex	0.825	0.654/1.042	0.107	0.991	0.705/1.393	0.958
BMI (kg/m2)	0.832	0.650/1.064	0.142	1.201	0.847/1.703	0.304
Current smokers	0.954	0.755/1.205	0.693	0.803	0.574/1.122	0.198
Lung function						
FEV_1_/FVC	0.862	0.773/0.962	0.008	0.953	0.810/1.121	0.559
FEV_1_ %Ref	0.877	0.812/0.947	0.001	1.000	0.864/1.157	0.997
FVC %Ref	0.941	0.868/1.019	0.132	0.998	0.896/1.111	0.966
Symptoms						
mMRC dyspnea scale 0–1		Reference			Reference	
mMRC dyspnea scale 2	1.349	1.055/1.726	0.017	1.332	0.859/2.065	0.200
mMRC dyspnea scale 3	1.371	0.993/1.892	0.055	1.434	0.925/2.223	0.107
mMRC dyspnea scale 4	3.036	1.930/4.777	< 0.001	1.974	1.083/3.600	0.026
Cough	1.238	0.998/1.535	0.052	1.344	0.838/2.156	0.220
Sputum	1.396	1.126/1.731	0.002	1.160	0.765/1.759	0.485
Treatment						
Short-acting bronchodilators	1.665	1.323/2.096	< 0.001	1.072	0.774/1.485	0.677
Long-acting ß_2_-agonists (LABA)	1.037	0.813/1.324	0.768	0.857	0.612/1.199	0.368
Long-acting muscarinic antagonists (LAMA)	0.918	0.739/1.141	0.440	0.723	0.527/0.991	0.044
Inhaled corticosteroids (ICS)	1.460	1.120/1.903	0.005	0.965	0.694/1.343	0.834
Inhaled combination therapy (LABA+ICS)	1.200	0.956/1.505	0.116	1.397	0.988/1.974	0.058
Systemic corticosteroids	1.764	1.118/2.785	0.015	0.820	0.544/1.235	0.342
Comorbidities						
Asthma	1.271	0.965/1.675	0.088	0.997	0.676/1.472	0.988
Diabetes mellitus	1.122	0.836/1.507	0.433	1.185	0.782/1.796	0.424
Hypertension	1.108	0.869/1.413	0.408	0.852	0.610/1.190	0.348
Coronary heart disease	1.239	0.933/1.644	0.139	1.177	0.796/1.741	0.413
Heart failure	0.988	0.695/1.404	0.947	0.988	0.598/1.631	0.961
Cerebrovascular Insult (CVI)	0.975	0.509/1.868	0.940	2.073	1.288/3.337	0.003
Other						
Sports (at least twice a week)	1.062	0.830/1.360	0.630	0.609	0.388/0.956	0.031
Pulmonary rehabilitation	1.061	0.636/1.769	0.821	0.578	0.294/1.135	0.111
Hospitalization for any reason	1.701	1.021/2.833	0.041	0.939	0.593/1.487	0.788

When having had no exacerbation at the most recent visit, lower FEV_1_/FVC and FEV_1_ values showed a significant association with exacerbation as well as hospitalization for any reason and pronounced dyspnea at the most recent visit. Furthermore, prescription of short-acting bronchodilators, inhaled or systemic corticosteroids were significantly associated with exacerbation.

When already having had an exacerbation at the most recent visit, pronounced dyspnea and cerebrovascular insult were significantly associated with re-exacerbation. Physical activity and treatment with LAMAs seemed to play a significant protective role.

### Best subset model

Table 3 shows the best subset models for predictive factors associated with exacerbation and re-exacerbation based on Akaike’s information criterion (AIC) [30]. We selected the following factors to design a best subset model: age, gender, BMI, current smoking history, FEV_1_ and FVC in percentage of reference values, respiratory symptoms (sputum, cough and dyspnea measured by mMRC dyspnea scale), hospitalization for any reason and main comorbidities (asthma, coronary heart disease, heart failure, hypertension and diabetes mellitus).

**Table 3:** Best subset models, multivariate Cox-regression

Factors	HR	SE	p
Best exacerbation subset model			
FEV_1_ %Ref	0.889	0.0414	0.0046
Sputum	1.439	0.1492	0.0150
Best re-exacerbation subset model			
Coronary heart disease	1.567	0.226	0.047
Hospitalization for any reason	0.583	0.306	0.078

Higher FEV_1_ values significantly reduced the probability of exacerbation when not having had an exacerbation at the most recent visit, whereas sputum increased probability of experiencing an exacerbation until next visit.

The best subset model for re-exacerbation included previous hospitalization and coronary heart disease. While coronary heart disease increased risk of re-exacerbation significantly, hospitalization at the most recent visit seemed to reduce risk for re-exacerbation in the best subset model.

## Discussion

With this multicenter, prospective, primary care cohort study, we were able to characterize a primary care COPD population and we were able to assess risk factors associated with exacerbation and re-exacerbation. In particular, lung function parameters (lower FEV_1_/FVC and lower FEV_1_) and symptoms such as pronounced dyspnea (in particular mMRC grade 4) or sputum production showed a significant association with exacerbation in a univariate analysis, whereas of these factors only pronounced dyspnea was significantly associated with re-exacerbation. Furthermore, prescription of short-acting bronchodilators, inhaled or systemic corticosteroids was significantly associated with exacerbation. We considered short-acting bronchodilator and corticosteroid prescription in this context most likely as surrogate markers of disease or symptom severity.

Using a multivariate Cox-regression model, formerly described in patients with Crohn’s disease, we were able to generate best subset models consisting of only two factors [24, 25]. Taking FEV_1_-values and sputum into account, seemed to be most relevant in this primary care setting, when assessing the transition from having no exacerbation to having an exacerbation. These findings underline the importance of regular symptom and spirometric assessment in primary care even in so-called “stable disease”.

Finally, re-exacerbation was significantly associated with pronounced dyspnea and patients having had a cerebrovascular insult or being less physically active. These results could partly support a benefit from pulmonary and physical rehabilitation. On the other hand, treatment with long-acting anticholinergics seemed to play a significant protective role, which seems reasonable. The best subset model for re-exacerbation included only previous hospitalization for any reason and coronary heart disease. Whilst coronary heart disease increased risk of re-exacerbation significantly, hospitalization at last visit seemed to reduce risk for re-exacerbation in the best subset model. An explanation for these findings could be that patients with comorbidities are more likely to experience frequent exacerbations [31] and that these patients might possibly benefit from in-patient treatment to stabilize their condition. However, it is still counterintuitive to some extent that overall hospitalization is on the other hand strongly associated with exacerbation in the univariate analysis.

Baseline characteristics of our cohort were similar in comparison with Norwegian, Swedish, Canadian and UK primary care cohorts [32–35]. However, with regards to GOLD severity grades, our patient population seemed to have more severe disease compared to the cohorts described by Wurst and colleagues (UK) or Sundh and colleagues (Sweden) [32, 34]. In accordance with the findings of Green et al. our analysis confirms the high prevalence of comorbidities, in contrast to their findings we found even higher rates of cardiac comorbidities [33].

The risk factors associated with exacerbation and re-exacerbation are largely in accordance with the literature [18, 36, 37], however the association of prescription of short-acting bronchodilators, inhaled or systemic corticosteroids was perhaps surprising, as some large trials have shown significant decrease in exacerbation risk with the use of inhaled steroids [38, 39]. However, a recent study of Magnussen et al. showed a similar risk of moderate or severe exacerbations for patients discontinuing inhaled glucocorticoids compared to patients who continued inhaled glucocorticoid treatment [40]. In addition, similar findings have been described in the Bergen COPD Cohort Study, a study which was designed to assess the ability of COPD characteristics and systemic inflammatory markers to predict the risk for acute COPD exacerbation frequency and duration [41]. Taking this into account, we suppose, as mentioned above, the prescription of these drugs to be surrogate markers for disease or symptom severity.

Concerning the prediction of exacerbation or hospital readmissions, some studies showed the value of other factors such as short acting bronchodilator use [42] or four meter gait speed, which we did not assess for in our cohort [43]. Furthermore, various new predictive scores such as the CODEX Index, the SCOPEX and the re-AE INDEX were established, which could be useful also in a primary care setting [19–21]. Unfortunately we were not able to test the SCOPEX, which seems to be easily applicable in primary care, as we have not recorded daily reliever use in our study population so far. Some recent research underlined the value of clinical symptoms and questioned the sensitivity of airflow limitation to diagnose smoking induced disease [44, 45]. Even though our best subset model for exacerbation highlights the importance of FEV_1_, which furthermore is in accordance with recently analyzed large data sources [46], we do agree that clinical symptoms do not only determine the degree of suffering of the patient but are a valuable and easy tool to evaluate the course of disease in primary care. The number of patients included in our cohort who did not fulfill a spirometric COPD diagnosis highlights the existence of patients with important clinical burden not fulfilling formal diagnostic criteria.

### Limitations and strengths of this study

This study has some limitations. Firstly, for reasons of anonymity, patient information was captured with questionnaires filled out by treating physicians. Therefore, it was not possible to gather further information by retrospective chart review, which would have been useful for the generation of a possibly more accurate exacerbation model. Secondly, as GPs participated on a voluntary basis and were not chosen in a randomized way, a certain overrepresentation of GPs interested in respiratory medicine might exist. However, given the similarity to other study populations, we believe that our patient sample is representative of the primary care setting. Thirdly, our cohort included few patients with GOLD 1 stage of disease and the mean follow up period was quite short.

However, we believe that this study has considerable strengths. The prospective, multicenter design including different GPs from various regions in Switzerland ensures representative, observational data from a primary care setting. Compared to other studies including patients with admission for AECOPD [20, 21] or excluding patients with asthma [19] our study population seems closer to a real life miscellaneous primary care population with a priori “stable” disease. Since we only included data from patients with a spirometric diagnosis of COPD for univariate and multivariate analysis, our findings should be accurate and to some extent generalizable in a primary care setting. Although the chosen model for multivariate analysis seems to have been used for the first time in COPD, which could be a limitation, it allows for relevant analysis and seems to be adequate for the present data structure and patient population.

### Implications of this study

Our data confirmed the existence of a non-negligible number of patients with a clinical diagnosis of COPD not fulfilling spirometric criteria and we think that these patients are not covered enough by current guidelines. We therefore see a need for further research and a greater academic awareness concerning diagnostic procedures and symptom relief in patients suffering from chronic respiratory symptoms with normal spirometry and patients suffering from multifactorial dyspnea. Clearly simple diagnostic tools to better characterize and ultimately improve treatment for patients with respiratory symptoms in primary care are needed. Some recently described predictive scores might help physicians in daily practice. Furthermore, we believe in the usefulness of a multistate model approach to better describe and ultimately improve prediction of course of disease in patients suffering from COPD, which would not only help doctors but health policy makers as well. “Big data” and new innovative ways to generate personalized health care data could be helpful to better characterize different phenotypes and personalize treatment accordingly. Meanwhile, we firmly believe in the utility of primary care based cohort studies in making real life data of our patients available for systematic evaluation.

## Conclusions

Our study confirmed well-established risk factors for AECOPD and confirmed the importance of spirometry in primary care, not only for the diagnosis of COPD in accordance with current guidelines but also as an important tool in order to improve treatment and reduce the risk for future exacerbations. Therefore, spirometry together with clinical assessment and medical history taking should build the basis for evaluating COPD patients in primary care. However, there is an ongoing debate on the sensitivity of spirometry as a diagnostic tool and our data revealed a substantial number of primary care patients suffering from respiratory symptoms whilst not fulfilling diagnostic spirometric criteria for COPD. Treatment for this patient collective remains challenging to physicians in their daily life and treatment guidelines probably do not reflect these circumstances adequately yet. Even if it is fairly difficult to perform trials in a primary care setting, we believe that further research is needed and more specifically elaborated treatment guidelines taking the circumstances of primary care into account.

## Abbreviations

AECOPD: Acute exacerbation of chronic obstructive pulmonary disease
AIC: Aikake information criterion
BMI: Body mass index
CODEX: Comorbidity, obstruction, dyspnea and previous severe exacerbations index
COPD: Chronic obstructive pulmonary disease
CVI: Cerebrovascular insult
DALY: Disability-adjusted life years
EKBB: Ethikkommission beider Basel
EKNZ: Ethikkommission Nordwest- und Zentralschweiz
FEV_1_: Forced expiratory volume in one second
FVC: Forced vital capacity
FEV_1_/FVC: FEV to FVC-ratio
GOLD: The Global Initiative for Chronic Obstructive Lung Disease
GP: General practitioner
HR: Hazard ratio
ICS: Inhaled corticosteroids
LABA: Long-acting ß_2_ agonists
LAMA: Long-acting muscarinic antagonists
mMRC: Modified Medical Research Council (dyspnea scale)
re-AE INDEX: Re-exacerbation index
SCOPEX: Score to predict short-term risk of COPD exacerbations
SD: Standard deviation
UK: United Kingdom
WHO: World health organization

## References

[1] Gershon AS, Warner L, Cascagnette P, Victor JC, To T (2011). Lifetime risk of developing chronic obstructive pulmonary disease: a longitudinal population study. Lancet.

[2] Chronic obstructive pulmonary disease among adults--United States, 2011. MMWR Morb Mortal Wkly Rep. 2012;61(46):938–43. 23169314

[3] Leuppi JD, Miedinger D, Chhajed PN, Buess C, Schafroth S, Bucher HC (2010). Quality of spirometry in primary care for case finding of airway obstruction in smokers. Respiration.

[4] World Health Organization (2004). The world health report : 2004 : changing history.

[5] World Health Organization (2008). World health statistics 2008.

[6] Srivastava K, Thakur D, Sharma S, Punekar YS (2015). Systematic review of humanistic and economic burden of symptomatic chronic obstructive pulmonary disease. PharmacoEconomics.

[7] Lopez AD, Shibuya K, Rao C, Mathers CD, Hansell AL, Held LS (2006). Chronic obstructive pulmonary disease: current burden and future projections. Eur Respir J.

[8] Salvi S (2014). Tobacco smoking and environmental risk factors for chronic obstructive pulmonary disease. Clin Chest Med.

[9] Jochmann A, Neubauer F, Miedinger D, Schafroth S, Tamm M, Leuppi JD. General practitioner’s adherence to the COPD GOLD guidelines: baseline data of the Swiss COPD cohort study. Swiss Med Wkly. 2010;140. 10.4414/smw.2010.13053. 10.4414/smw.2010.13053 20407960

[10] Jochmann A, Scherr A, Jochmann DC, Miedinger D, Torok SS, Chhajed PN (2012). Impact of adherence to the GOLD guidelines on symptom prevalence, lung function decline and exacerbation rate in the Swiss COPD cohort. Swiss Med Wkly.

[11] Abu Hussein N, Ter Riet G, Schoenenberger L, Bridevaux PO, Chhajed PN, Fitting JW (2014). The ADO index as a predictor of two-year mortality in general practice-based chronic obstructive pulmonary disease cohorts. Respiration.

[12] Sullivan SD, Ramsey SD, Lee TA (2000). The economic burden of COPD. Chest.

[13] Kanner RE, Anthonisen NR, Connett JE (2001). Lung health study research G. lower respiratory illnesses promote FEV(1) decline in current smokers but not ex-smokers with mild chronic obstructive pulmonary disease: results from the lung health study. Am J Respir Crit Care Med.

[14] Donaldson GC, Seemungal TA, Bhowmik A, Wedzicha JA (2002). Relationship between exacerbation frequency and lung function decline in chronic obstructive pulmonary disease. Thorax.

[15] Seemungal TA, Donaldson GC, Paul EA, Bestall JC, Jeffries DJ, Wedzicha JA (1998). Effect of exacerbation on quality of life in patients with chronic obstructive pulmonary disease. Am J Respir Crit Care Med.

[16] Makris D, Moschandreas J, Damianaki A, Ntaoukakis E, Siafakas NM, Milic Emili J (2007). Exacerbations and lung function decline in COPD: new insights in current and ex-smokers. Respir Med.

[17] Soler-Cataluna JJ, Martinez-Garcia MA, Roman Sanchez P, Salcedo E, Navarro M, Ochando R (2005). Severe acute exacerbations and mortality in patients with chronic obstructive pulmonary disease. Thorax.

[18] Hurst JR, Vestbo J, Anzueto A, Locantore N, Mullerova H, Tal-Singer R (2010). Susceptibility to exacerbation in chronic obstructive pulmonary disease. N Engl J Med.

[19] Make BJ, Eriksson G, Calverley PM, Jenkins CR, Postma DS, Peterson S (2015). A score to predict short-term risk of COPD exacerbations (SCOPEX). Int J Chronic Obstructive pulmonary Dis.

[20] Almagro P, Soriano JB, Cabrera FJ, Boixeda R, Alonso-Ortiz MB, Barreiro B (2014). Short- and medium-term prognosis in patients hospitalized for COPD exacerbation: the CODEX index. Chest.

[21] Liu D, Peng SH, Zhang J, Bai SH, Liu HX, Qu JM (2015). Prediction of short term re-exacerbation in patients with acute exacerbation of chronic obstructive pulmonary disease. Int J Chronic obstructive pulmonary dis..

[22] Donaldson GC, Law M, Kowlessar B, Singh R, Brill SE, Allinson JP, et al. Impact of prolonged exacerbation recovery in chronic obstructive pulmonary disease. Am J Respir Crit Care Med. 2015;192(8):943-50. 10.1164/rccm.201412-2269OC. 10.1164/rccm.201412-2269OC PMC4642208 26151174

[23] Westerik JA, Metting EI, van Boven JF, Tiersma W, Kocks JW, Schermer TR (2017). Associations between chronic comorbidity and exacerbation risk in primary care patients with COPD. Respir Res.

[24] Silverstein MD, Loftus EV, Sandborn WJ, Tremaine WJ, Feagan BG, Nietert PJ (1999). Clinical course and costs of care for Crohn's disease: Markov model analysis of a population-based cohort. Gastroenterology.

[25] Therneau TM, Grambsch PM (2000). Modeling survival data: extending the cox model.

[26] Standardization of Spirometry, 1994 Update (1995). American Thoracic Society. Am J Respir Crit Care Med.

[27] Miller MR, Hankinson J, Brusasco V, Burgos F, Casaburi R, Coates A (2005). Standardisation of spirometry. Eur Respir J.

[28] Brandli O, Schindler C, Kunzli N, Keller R, Perruchoud AP (1996). Lung function in healthy never smoking adults: reference values and lower limits of normal of a Swiss population. Thorax.

[29] Bridevaux PO, Dupuis-Lozeron E, Schindler C, Keidel D, Gerbase MW, Probst-Hensch NM (2015). Spirometer replacement and serial lung function measurements in population studies: results from the SAPALDIA study. Am J Epidemiol.

[30] Akaike H (1974). New Look at Statistical-Model Identification. Ieee T Automat Contr.

[31] McGarvey L, Lee AJ, Roberts J, Gruffydd-Jones K, McKnight E, Haughney J (2015). Characterisation of the frequent exacerbator phenotype in COPD patients in a large UK primary care population. Respir Med.

[32] Wurst KE, Punekar YS, Shukla A (2014). Treatment evolution after COPD diagnosis in the UK primary care setting. PLoS One.

[33] Green ME, Natajaran N, O'Donnell DE, Williamson T, Kotecha J, Khan S (2015). Chronic obstructive pulmonary disease in primary care: an epidemiologic cohort study from the Canadian primary care sentinel surveillance network. CMAJ open.

[34] Sundh J, Osterlund Efraimsson E, Janson C, Montgomery S, Stallberg B, Lisspers K (2013). Management of COPD exacerbations in primary care: a clinical cohort study. Primary care respiratory journal : journal of the General Practice Airways Group.

[35] Al-ani S, Spigt M, Hofset P, Melbye H (2013). Predictors of exacerbations of asthma and COPD during one year in primary care. Fam Pract.

[36] Mullerova H, Shukla A, Hawkins A, Quint J (2014). Risk factors for acute exacerbations of COPD in a primary care population: a retrospective observational cohort study. BMJ Open.

[37] Niewoehner DE, Lokhnygina Y, Rice K, Kuschner WG, Sharafkhaneh A, Sarosi GA (2007). Risk indexes for exacerbations and hospitalizations due to COPD. Chest.

[38] Calverley PM, Anderson JA, Celli B, Ferguson GT, Jenkins C, Jones PW (2007). Salmeterol and fluticasone propionate and survival in chronic obstructive pulmonary disease. N Engl J Med.

[39] Yang IA, Clarke MS, Sim EH, Fong KM (2012). Inhaled corticosteroids for stable chronic obstructive pulmonary disease. The Cochrane database of systematic reviews.

[40] Magnussen H, Disse B, Rodriguez-Roisin R, Kirsten A, Watz H, Tetzlaff K (2014). Withdrawal of inhaled glucocorticoids and exacerbations of COPD. N Engl J Med.

[41] Husebo GR, Bakke PS, Aanerud M, Hardie JA, Ueland T, Gronseth R (2014). Predictors of exacerbations in chronic obstructive pulmonary disease--results from the Bergen COPD cohort study. PLoS One.

[42] Jenkins CR, Postma DS, Anzueto AR, Make BJ, Peterson S, Eriksson G (2015). Reliever salbutamol use as a measure of exacerbation risk in chronic obstructive pulmonary disease. BMC pulmonary medicine.

[43] Kon SS, Jones SE, Schofield SJ, Banya W, Dickson MJ, Canavan JL, et al. Gait speed and readmission following hospitalisation for acute exacerbations of COPD: a prospective study. Thorax. 2015;70(12):1131-7. 10.1136/thoraxjnl-2015-207046. Epub 2015 Aug 17. 10.1136/thoraxjnl-2015-207046 26283709

[44] Woodruff PG, Barr RG, Bleecker E, Christenson SA, Couper D, Curtis JL (2016). Clinical significance of symptoms in smokers with preserved pulmonary function. N Engl J Med.

[45] Fabbri LM (2016). Smoking, not COPD, as the disease. N Engl J Med.

[46] Hoogendoorn M, Feenstra TL, Boland M, Briggs AH, Borg S, Jansson SA, et al. Prediction models for exacerbations in different COPD patient populations: comparing results of five large data sources. Int J Chron Obstruct Pulmon Dis. 2017;12:3183–94. 10.2147/COPD.S142378 PMC5677310 29138546

